# Establishment of a Luciferase-Based Reporter System to Study Aspects of Human Cytomegalovirus Infection, Replication Characteristics, and Antiviral Drug Efficacy

**DOI:** 10.3390/pathogens13080645

**Published:** 2024-07-31

**Authors:** Julia Tillmanns, Jintawee Kicuntod, Antonia Ehring, Endrit Elbasani, Eva Maria Borst, Debora Obergfäll, Regina Müller, Friedrich Hahn, Manfred Marschall

**Affiliations:** 1 Institute for Clinical and Molecular Virology, Friedrich-Alexander University of Erlangen-Nürnberg (FAU), 91054 Erlangen, Germany; jul.tillmanns@fau.de (J.T.); jintawee.kicuntod@extern.uk-erlangen.de (J.K.); antonia.ehring@fau.de (A.E.); debora.obergfaell@fau.de (D.O.); mueller.regina@uk-erlangen.de (R.M.); friedrich.hahn@uk-erlangen.de (F.H.); 2 Institute of Virology, Hannover Medical School (MHH), 30625 Hannover, Germany; elbasani.endrit@mh-hannover.de (E.E.); borst.eva@mh-hannover.de (E.M.B.)

**Keywords:** medically important viral pathogens, human cytomegalovirus (HCMV), viral replication characteristics and efficiency, antiviral drug development, firefly luciferase reporter (FLuc), recombinant HCMV TB40-FLuc, infection of primary human cultured cells, advantages of the reporter system, future applications in molecular HCMV analysis

## Abstract

Human cytomegalovirus (HCMV) represents a highly medically important pathogen which has constantly been the subject of both molecular and clinical investigations. HCMV infections, especially those in high-risk patients, still raise many unanswered questions, so current investigations are focused on viral pathogenesis, vaccine development, and options for antiviral drug targeting. To this end, the use of suitable viral strains as well as recombinant reporter constructs in cultured cells and model systems has specific significance. We previously reported on the application of various herpesviruses that express green, red, or related fluorescent proteins, especially in the fields of virus–host interaction and antiviral research. Here, we characterized a recombinant version of the clinically relevant and cell type-adaptable HCMV strain TB40, which expresses firefly luciferase as a quantitative reporter of viral replication (TB40-FLuc). The data provide evidence for five main conclusions. First, HCMV TB40-FLuc is employable in multiple settings in primary human cells. Second, viral reporter signals are easily quantifiable, even at early time points within viral replication. Third, the FLuc reporter reflects the kinetics of viral intracellular replication, cascade-like viral IE-E-L protein production, and progeny release. Fourth, as relates to specific applications of the TB40-FLuc system, we demonstrated the reliability of quantitative antiviral compound determination in multi-well formats and its independence from fluorescence-based measurements in the case of autofluorescent inhibitors. Finally, we illustrated increased reporter sensitivity in comparison to other recombinant HCMVs. In essence, recombinant HCMV TB40-FLuc combines several molecular properties that are considered beneficial in studies on viral host tropism, replication efficiency, and antiviral drug assessment.

## 1. Introduction

Human cytomegalovirus (HCMV) is a member of the β-subfamily of *Herpesviridae* and shows a worldwide distribution. HCMV infection persists life-long in the human host and is mostly asymptomatic in immunocompetent individuals. In immunosuppressed and immunonaïve hosts such as fetuses or infants, however, HCMV may cause severe or even life-threatening sequelae, and HCMV itself may further weaken immune responses [1,2,3]. Specifically, congenital HCMV infection (cCMV) acquired during pregnancy still represents a serious medical problem, frequently leading to developmental defects, particularly upon generalized HCMV infection. Viral reactivation and reinfection may occasionally occur, both mostly asymptomatic or accompanied by mild febrile illness. So far, no vaccine has been approved for the prevention of HCMV infections, and although anti-HCMV drugs are available, problems including unwarranted side-effects or drug-induced viral resistance mutations interfere with the success of therapy, and the mechanistic repertoire of approved drugs is still limited [4,5,6]. This underlines the need for novel antiviral strategies, accessible target proteins, and viral analysis systems.

HCMV-induced pathogenesis is widely determined by the magnitude of viral replication [2]. Pathogenic HCMV infection is complex and there are three distinct infection scenarios [7]. Primary infection occurs when an individual with no immunity against this virus becomes infected for the first time. Upon the establishment of viral latency, HCMV may reactivate as a secondary infection. Third, HCMV reinfection may occur via superinfection with a different virus strain in the absence of strain-specific immunity [7,8]. In all cases, the pathogenesis of the infection is frequently correlated with viral load in tissues and the overall productive level of HCMV replication. Accordingly, the antiviral treatment strategy of preemptive therapy is based on a close-meshed diagnostic inspection of patients, and the therapeutic decision is frequently linked to an increase in viral load [9]. In the context of the molecular analysis of factors determining HCMV productivity and lytic replication, specific model systems in vivo and in vitro have been established in great detail [10,11,12]. In particular, the clinically relevant strain TB40 is of interest, because this strain shows adaptable permissiveness across different cell types, i.e., with productive replication in both epithelial/endothelial cells and/or fibroblasts. Our earlier regulatory investigations demonstrated that TB40 comprises several parameters similar or identical to clinical isolates of HCMV, and proved to possess very valuable applicability for the analysis of viral phenotypes upon site-directed mutagenesis and the generation of viral BAC recombinants [11]. Specifically, a number of clinically relevant viral strains have been subjected to comparative genetic studies [12]. Their recombinant reporter constructs in cultured cells and model systems have been investigated for parameters of viral replication and antiviral drug sensitivity [13,14,15,16]. Thus, the use of HCMV TB40 as a reporter strain has offered an opportunity to characterize its distinct properties as displayed in various molecular analyses in comparison with further reference strains.

In the present study, we generated a luciferase-expressing HCMV reporter (TB40-FLuc) to study viral replication characteristics in cultured cell models, including productivity- and pathogenesis-relevant viral determinants, as well as antiviral drug efficacy. The results demonstrated that HCMV TB40-FLuc is employable in multiple settings in primary human cells as an easy mode of reporter signal quantitation, analysis of viral intracellular replication, and the assessment of antiviral agents which include autofluorescent compounds. Thus, the recombinant viral model system may be beneficial for a number of different applications in the molecular analysis of HCMV replication.

## 2. Materials and Methods

### 2.1. Genetic Recombination and Reconstitution of Infectious HCMV TB40-FLuc

The HCMV mutant TB40-FLuc was generated by en passant mutagenesis using TB40 BAC4 as a backbone [17], with a specific replacement of open reading frame (ORF) RL13 by the ORF coding for firefly luciferase (FLuc), set under transcriptional control of a minimal version of the murine cytomegalovirus (MCMV) major immediate early promoter–enhancer (MIEP). Firstly, pMCMV3-KnR-FLuc was constructed as template for mutagenesis by inserting the FLuc sequences into pMCMV3 containing the MCMV MIEP and the SV40 polyadenylation signal [18]. To this end, the primer pair of GB-pMCMV3-FLuc.fw (5′-gagctcctcgctgcaggccaccatggaagacgccaaaaac-3′) and GB-pMCMV3-FLuc.rv (5′-gcaataaacaagttaacgtcgacttacacggcgatctttcc-3′) was used to amplify the ORF FLuc from plasmid pGL3-Basic (Promega, Madison, WI, USA), and the resulting PCR fragment was cloned into the PstI and SmaI restriction sites of pMCMV3 employing the Gibson Assembly master mix following the manufacturer’s recommendations (New England Biolabs, Ipswich, MA, USA, cat no. E2611S), giving rise to pMCMV3-FLuc. Subsequently, a kanamycin resistance cassette comprising all elements necessary for en passant mutagenesis [19] was amplified from pori6K-RIT [20] with the primers PstI-B-KnR.fw (5′-gggctgcaggccaccatggaagacgcgacgcatcgtggccggatctc-3′) and PstI-A-KnR.rv (5′-gggctgcagcgaggagctctgcgttcgtgaccacgtcgtggaatgc-3′) and inserted into the PstI restriction site of pMCMV3-FLuc, yielding pMCMV3-KnR-FLuc. For en passant mutagenesis, a PCR product harboring the FLuc plus KnR sequences was generated from pMCMV3-KnR-FLuc utilizing the primer pair of TB40-RL13-MIEP.fw (5′-acaacatccgaagaaacatcaatgcccattaaccgaaatccaacaacgtttaaacggtactttcccatagctg-3′) and TB40-RL13-FLuc.rv (5′-gaaacatattattggctaaaaagaaaagcaaaagtttattggtgtgcatgaagttaacgtcgacttacacgg-3′) and was inserted into the TB40 BAC4 by homologous recombination in *E. coli*. The KnR sequences were then removed as described earlier [19]. The resulting BAC TB40-FLuc was used to reconstitute mutant virus by adenofection [21] of hTERT-RPE-1 cells (Clontech, Mountain View, CA, USA).

### 2.2. Cell Culture and Virus Infection

Primary human foreskin fibroblasts (HFFs, own repository of primary cell cultures) and human retinal pigment epithelial ARPE-19 cells (ATCC, Manassas, VA, USA) were maintained at 37 °C, 5% CO_2_, and 80% humidity. As the cultivation medium, Eagle’s minimal essential medium was used (MEM; 21090055, Thermo Fisher Scientific, Waltham, MA, USA), supplemented with 10% fetal bovine serum (AC-SM-0027, anprotec, Bruckberg, Germany), 1 × GlutaMAX^TM^ (35050038, Thermo Fisher Scientific, Waltham, MA, USA), and 10 µg/mL gentamicin (22185.03, SERVA, Heidelberg, Germany). HFFs were infected either with HCMV strains AD169, variant UK, or TB40 in their wild-type versions (WT) [22] or with recombinant viruses AD169-GFP [16], TB40-YFP [11,23], or TB40-FLuc at suitable multiplicities of infection (MOI). Depending on whether single- or multi-round analyses of viral infection were performed, samples were collected at appropriate time points post-infection (p.i.).

### 2.3. Luminescence-Based and Fluorescence-Based HCMV Replication Assays

To analyze the antiviral activity of the compounds, HFFs were seeded one day prior to infection in a 96-well format (1.35 × 10^4^ cells per well). After infection with HCMV reporter strains AD169-GFP or TB40-FLuc for 90 min at 37 °C, serial dilutions of compounds were added. Virus dilutions were adjusted to 25–40% of infected cells at 7 days (d) p.i. (AD169-GFP, TB40-YFP) or 12 d p.i. (TB40-FLuc). For analysis, AD169-GFP- or TB40-YFP-infected cells were fixed with 10% formalin for 10 min at room temperature, followed by washing with PBS and automated fluorescence quantitation in PBS using the Victor X4 microplate reader (PerkinElmer, Waltham, MA, USA) or the ImageXpress^®^ Pico device (Molecular Devices LLC, San Jose, CA, USA). For luminescence-based approaches, a standard assay buffer containing 100 mM phosphate buffer (an appropriate combination of 1M KH_2_PO_4_ and 1M K_2_HPO_4_ to reach pH 7.8) and 15 mM MgSO_4_ was prepared. TB40-FLuc-infected cells were lysed using a 100 µL/well standard assay buffer which additionally contained 1% Triton X-100. For automated luminescence quantitation, the standard assay buffer was supplemented with 10 mM rATP (Abcam, Cambridge, UK; dissolved in H_2_O, adjusted to pH 7) and 1 mM luciferin (PJK GmbH, Kleinbittersdorf, Germany; dissolved in standard assay buffer). Thereafter, 50 µL of prepared buffer was freshly added to each cell lysate prior to measurement of the luminescent signal by utilizing a Victor X4 microplate reader.

### 2.4. Neutral Red Assay

To determine the cytotoxic effects of the compounds, HFFs were seeded in a 96-well format (1.35 × 10^4^ HFFs per well) one day prior to treatment with the indicated compounds. Examination of cell viability was performed after an incubation time identical with the corresponding antiviral assay. Assay conditions were as described previously [24] using 40 µg/mL Neutral Red solution (Sigma-Aldrich, St. Louis, MO, USA). Dye incorporation was determined by fluorescence measurement at 560/630 nm for excitation/emission, respectively, in a Victor X4 microplate reader (PerkinElmer, Waltham, MA, USA).

### 2.5. Quantitative Real-Time PCR (qPCR)

For the determination of viral loads in infected cell culture supernatants, the HCMV-specific quantitative PCR was performed as described previously [25]. HFFs were infected with either TB40-FLuc, TB40-WT, or AD169-WT with equal MOIs and samples were harvested from the cell supernatant at 1–5 d p.i. For isolation of viral DNA, the collected supernatants were digested with proteinase K (Sigma-Aldrich, St. Louis, MO, USA) at 56 °C for 1 h, followed by sample denaturation at 95 °C for 5 min. The real-time qPCR, using TaqMan PCR Mastermix (Applied Biosystems, Waltham, MA, USA), targeted a gene region within the major immediate early gene region exon 4 via the two primers 5′-AAGCGGCCTCTGATAACCAAG-3′ and 5′-GAGCAGACTCTCAGAGGATCG-3′ for sequence-specific annealing and a probe (5′-CATGCAGATCTCCTCAATGCGCGC-3′) labeled with 6-carboxyfluorescein reporter dye and 6-carboxytetramethylrhodamine quencher dye.

### 2.6. Indirect Immunofluorescence (IF) Analysis and Confocal Laser-Scanning Microscopy

In order to investigate viral protein expression and localization, 3 × 10^5^ HFFs were seeded in 6-well plates one day prior to infection with TB40-FLuc (MOI 1). At 1, 3, or 5 d p.i., the infected cells were fixed with 10% formalin and were subjected to IF staining as described previously [26]. The primary and secondary antibodies used for staining were mAb-IE1 p63-27 (own repository; [27]), mAb-UL44 (BS 510) (kindly provided by B. Plachter, Mainz, Germany), mAb-MCP (28-4) (kindly provided by W. Britt, Birmingham, AL, USA), mAb-lamin A/C (ab108595, Abcam, Cambridge, UK), mAb-anti-CMV (IE1) Alexa 488 (MAb810X, Merck KGaA, Darmstadt, Germany), anti-mouse IgG Alexa 488 (A11029), and anti-rabbit IgG Alexa 555 (A21429; final two from Thermo Fisher Scientific, Waltham, MA, USA). Analysis was performed using a TCS SP5 confocal laser-scanning microscope with Leica LAS AF software version 1.8.2-1465 (Leica Microsystems, Wetzlar, Germany). Images were further processed using Photoshop CS5 (Adobe Inc., San José, CA, USA).

### 2.7. SDS Polyacrylamide Gel Electrophoresis (SDS-PAGE) and Western Blot Analysis

HFFs were seeded in 6-well plates at a density of 3 × 10^5^ cells/well one day prior to infection with HCMV TB40-FLuc, TB40-WT, or AD169-WT (MOI 0.5-1). SDS-PAGE and standard Western blot analysis were performed using total cell lysates at equal protein amounts as described previously [28]. The antibodies used for staining were mAb-IE1 (p63-27; see Section 2.6), mAb-UL44 (BS 510; see Section 2.6), mAb-MCP (28-4; see Section 2.6), and mAb-β-actin (A5441; Sigma-Aldrich, St. Louis, MO, USA).

### 2.8. Antiviral Compounds

Antiviral compounds were derived from the following sources: ganciclovir (GCV; Sigma-Aldrich, St. Louis, MO, USA), maribavir (MBV; MedChemExpress, Monmouth Junction, NJ, USA), letermovir (LMV; Ambeed, Arlington Heights, IL, USA), merbromin (MBM; M7011, Sigma-Aldrich, St. Louis, MO, USA), acyclovir (ACV; MedChemExpress, Monmouth Junction, NJ, USA), cidofovir (CDV; MedChemExpress, Monmouth Junction, NJ, USA), brincidofovir (BCV; MedChemExpress, Monmouth Junction, NJ, USA), and MSC2530818 (MSC; Sigma-Aldrich, St. Louis, MO, USA). All drugs were dissolved in DMSO and stored at −20 °C.

## 3. Results and Discussion

### 3.1. Generation of Recombinant HCMV BAC and Reconstitution of the TB40-FLuc Reporter Virus

**Figure 1:**
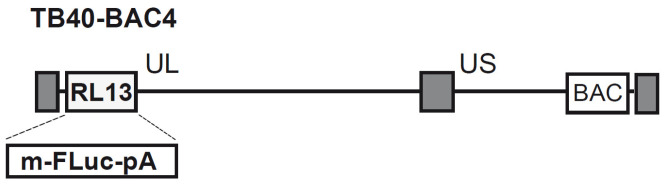
Generation of a TB40-based recombinant HCMV bacterial artificial chromosome (BAC) encoding firefly luciferase. The FLuc expression cassette driven by the murine cytomegalovirus-specific MIEP (m) together with the simian virus 40-specific polyadenylation signal (pA) was introduced into TB40 BAC4 [17], replacing the RL13 locus of the UL segment of the viral genome. Infectious HCMV TB40-FLuc was reconstituted by transfection of hTERT-RPE-1 cells with the recombinant TB40-FLuc BAC, and was amplified by passaging culture media samples on fresh cells to produce a viral stock.

In order to broaden our repertoire of reporter constructs to study the replicative properties and antiviral drug sensitivity of cytomegaloviruses, we used the HCMV BAC of strain TB40 as the genetic basis [17]. Compared to highly passaged HCMV laboratory strains, such as AD169, TB40 preserved the ability to infect various cell types in addition to fibroblasts, e.g., epithelial and endothelial cells, macrophages, as well as dendritic cells. The ORF encoding firefly luciferase (FLuc), driven by MIEP sequences derived from murine cytomegalovirus, was employed to enable fast and highly sensitive detection of HCMV-infected cells. We decided to replace the HCMV ORF RL13 encoding of a highly glycosylated viral envelope protein [29,30] with the MIEP-FLuc sequences (Figure 1) because RL13 is non-essential in cell culture. Moreover, RL13 has a strong propensity to become inactivated by mutation upon passage in cell culture, as it acts as a repressor of viral replication [29,30,31]. Furthermore, its presence impairs the release of cell-free virus from infected cells, presumably together with other viral factors [32,33]. Following reconstitution of infectious progeny from the BAC TB40-FLuc, the resulting reporter virus was thoroughly characterized regarding luciferase expression, growth properties, and protein expression.

### 3.2. Establishment of the Infection System with HCMV TB40-FLuc in Primary Human Fibroblasts

**Figure 2:**
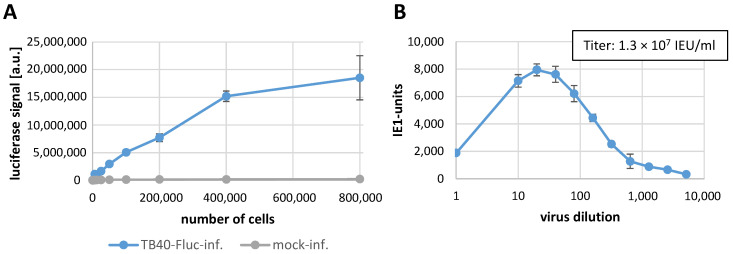
Titration of luciferase signal by HCMV TB40-FLuc infection. (**A**) HFFs were incubated with TB40-FLuc for 10 d, while uninfected cells served as a negative control. The cell number-dependent luciferase signal of lysed cells was measured by the Victor X4 microplate reader at 10 d p.i. in quadruplicates. (**B**) HFFs were cultivated in a 96-well plate and infected with undiluted virus stock or a dilution series, followed by treatment with 3 μM MBV to ensure limitation to a single round of viral replication. At 5 d p.i., cells were fixed and stained with an IE1-specific antibody for titer calculation. Measurements by the ImageXpress^®^ Pico device were performed in quadruplicate; samples and mean values ± SD of positive wells (IE1-units) are given. Abbreviations: a.u., arbitrary luciferase units; IEU/mL, immediate early protein-expressing units per ml of the virus stock solution.

In a first stage of establishment of the HCMV TB40-FLuc infection, we used primary human foreskin fibroblasts (HFFs) as a fully HCMV-permissive cultured cell model [16], and performed both serial increases of cell numbers per well and titration of the virus by luciferase signal measurement (Figure 2). At 10 d p.i., the luciferase reporter signal was determined in quadruplicates as a function of increasing cell numbers (Figure 2A) in the absence of any background signals obtained with a mock-infected control. Since the infected HFFs showed high intensities of luciferase signal, a 96-well plate format was subsequently used in the following step, in which stock virus dilutions were applied for titer determination. In this case, when applying 96-well plates, infected cells were treated with the antiviral drug maribavir (MBV, 3 μM) to limit viral replication to a single-round infection. MBV treatment was expected to inhibit the late stage of HCMV replication (i.e., a block in pUL97 kinase-dependent viral nuclear egress and subsequent virus release; [34,35]), so that viral progeny spread in the culture could be ruled out. At 5 d p.i. (thus a time interval that ensured single-round infection under MBV conditions), the quantity of virus positive cells per well was counted in order to determine the optimal inoculum dilution (i.e., positive for the marker of viral immediate early protein, as stained by indirect IF assay using an IE1-specific antibody). These values were set as the basis for calculation of the infectious virus titer (Figure 2B).

### 3.3. Replication Kinetics of HCMV TB40-FLuc in Single-Round or Multi-Round Settings

**Figure 3:**
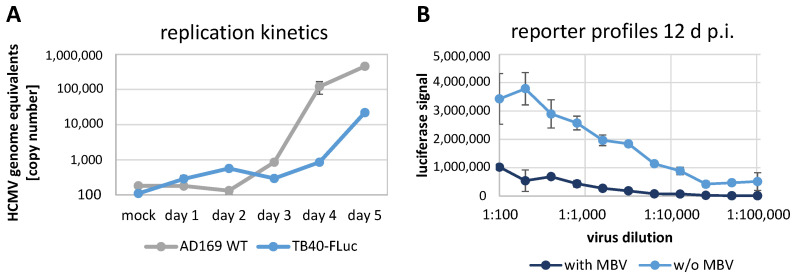
Viral replication kinetics and comparison of single-round/multi-round infection. (**A**) HFFs were cultivated in 6-well plates and infected with HCMV AD169-WT or TB40-Fluc at an MOI of 1 before samples were taken from the supernatants on 1–5 d p.i. The number of HCMV genome equivalents in the samples were analyzed by HCMV-specific qPCR, and uninfected cells (mock) served as a negative control. The qPCR measurements were performed in triplicate samples and mean values ± SD are given. (**B**) For a single-round versus multi-round comparison, HFFs were infected with a dilution series of TB40-FLuc followed by treatment with or without 3 μM MBV. At 12 d p.i., cells were lysed and luciferase signal was measured to compare these settings (mean values of quadruplicates ± SD).

According to the results described above, viral replication kinetics were determined for two different viral strains (Figure 3A). For this purpose, viral supernatant samples from infected cells were harvested from 1 to 5 d p.i. and subjected to a virus-specific qPCR for the determination of HCMV genome equivalents. The data curves indicated a start of virus release by 3 or 4 d p.i. for AD169-WT or TB40-FLuc, respectively, and a more rapid increase of AD169-WT until d 5 (Figure 3A, grey curve). The TB40-FLuc virus also showed an exponential phase of virus production until d 5 (Figure 3A, blue curve), but the kinetics appeared slightly delayed and the optima were eventually reached at later time points. For this reason, the virus infection experiments used for antiviral drug assessment (see Section 3.5), as performed in multi-round settings, were harvested at 12 d p.i. for TB40-FLuc and 7 d p.i. for AD169-WT or AD169-GFP (both equating to approx. 3 to 2.5 replicative rounds). Next, for the FLuc reporter virus, single-round and multi-round infections were performed in parallel to compare the profiles of reporter expression (Figure 3B). While the single-round setting (with MBV, dark blue) indicated that signals generally remained at very low levels, the multi-round infection (w/o MBV, light blue) showed a clear linear relationship between signal level and virus dilution. This result demonstrated that the system allows for the use of FLuc reporter measurements in quantitative terms within the linear range (1:200 to 1:20,000) of virus dilutions.

### 3.4. Characterization of Viral Protein Expression Patterns in HCMV TB40-FLuc-Infected Cells

**Figure 4:**
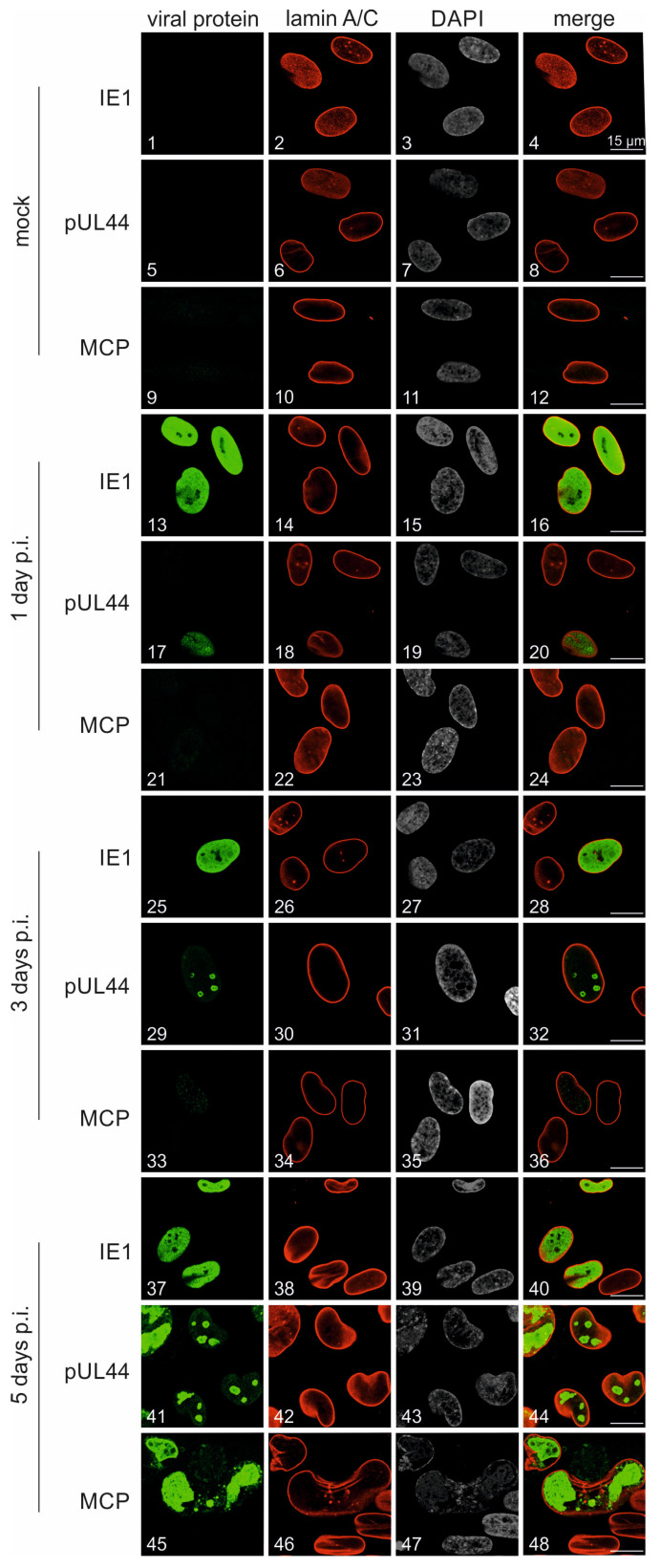
Confocal imaging of the viral cascade of IE-E-L protein production. HFFs were infected with TB40-FLuc at an MOI of 1 and fixed 1, 3, or 5 d p.i. Uninfected cells (mock) were fixed on d 5 and served as negative controls. IF staining was performed with viral protein-specific primary antibodies (mAb-IE1, detects immediate early IE1p72; mAb-UL44, detects early pUL44; mAb-MCP, detects late major capsid protein MCP) as well as lamin A/C antibody (mAb-Lamin A/C) staining for the nuclear envelope, and DAPI counterstaining indicating the nucleus. The results were imaged using a confocal laser-scanning microscope.

**Figure 5:**
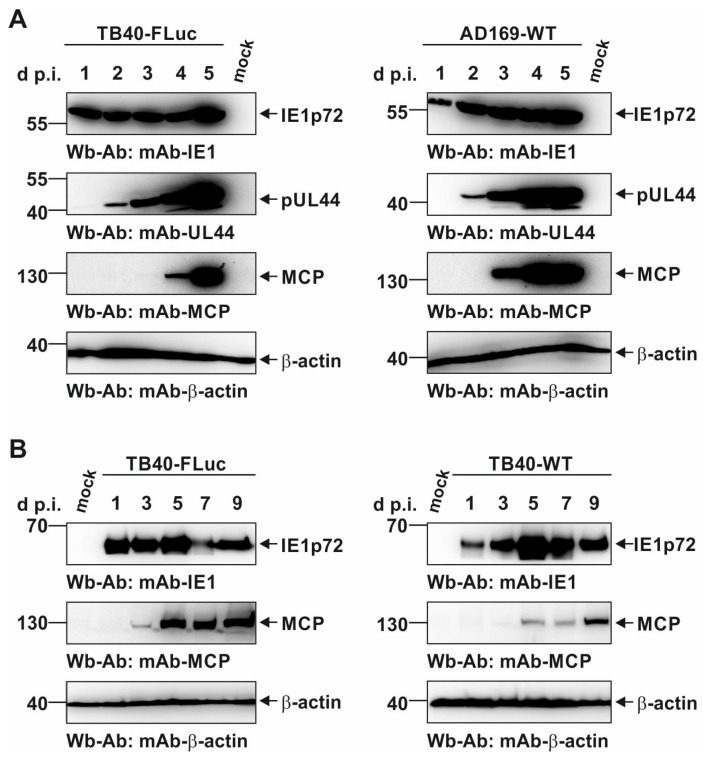
Kinetics of viral protein production of HCMV TB40-FLuc in comparison to AD169-WT or TB40-WT. (**A**) HFFs were infected with TB40-FLuc (**left**) or AD169-WT (**right**) at an MOI of 1, and uninfected cells (mock) served as a negative control. (**B**) Similarly, HFFs were infected with TB40-FLuc (**left**) or TB40-WT (**right**) at an MOI of 0.5 in order to compare viral protein production in the presence or absence of the FLuc reporter, respectively. After the indicated number of days, the infected cells were harvested before total lysates were prepared and subjected to standard Wb analysis using specific antibodies as indicated.

The expression time course and intracellular localization of viral proteins was characterized in FLuc-infected HFFs. To monitor the cascade-like expression pattern of the viral proteins IE1 (immediate early, IE), pUL44 (early, E), and MCP (late, L), an indirect immunofluorescence staining was performed and samples were analyzed by confocal imaging (Figure 4). The expression of IE1p72 was evenly spread throughout the nuclei (Figure 4, images 13, 25, 37), as indicated by the lamin A/C counterstaining used as a nuclear envelope marker. Viral pUL44 showed likewise nuclear accumulation, whereby expression started on 1 d p.i. (image 17), which was then followed by an increasingly clear appearance of its typical speckled pattern within viral replication compartments (VRCs; images 29, 41). Compared to this, expression of the true late viral marker MCP was not limited to the nucleus, but also included a partial cytoplasmic localization (image 45), indicating viral capsid nucleocytoplasmic egress. These findings demonstrated a basically normal pattern of HCMV-specific protein production and may indicate a slightly prolonged replication kinetic of the TB40-FLuc reporter strain (as compared with the established laboratory strain AD169-GFP; see Figure 5).

To demonstrate viral protein quantities and kinetics in a comparison between the novel reporter strain TB40-FLuc and the standard laboratory strain AD169-WT, an SDS-PAGE/Wb analysis was performed (Figure 5A, left panels and right panels, respectively). Each sample series in the course of 1–5 d p.i. was stained for three viral proteins (IE1p72, pUL44, MCP; β-actin was included as a housekeeping protein), and uninfected cells (mock) served as a negative control. As seen for all three viral proteins, expression patterns were mostly identical in quantitative terms, with the only difference being a later onset in MCP production by TB40-FLuc (compare Figure 5A, left panel MCP, lanes 4–5 d p.i., with right panel MCP, lanes 3–5 d p.i.). This result confirms the reliable production of the IE-E-L pattern of viral proteins in the case of TB40-FLuc compared to the reference strain AD169-WT.

A second comparison concerned the TB40-FLuc reporter strain in regard to its non-reporter parental strain TB40-WT. Here, an extended replication kinetic was monitored along 9 d p.i. (Figure 5B). The main focus was placed on the late markers of infection, i.e., the production of viral capsid protein (MCP), during this time range spanning approx. three rounds of viral replication. Notably, the course of MCP production, starting at 3 d p.i., was almost identical between the two viruses, and no substantial difference could be observed (Figure 5B, lanes 1–9 d p.i.). This result further supports the unaffected replication behavior of the HCMV reporter TB40-FLuc.

**Figure 6:**
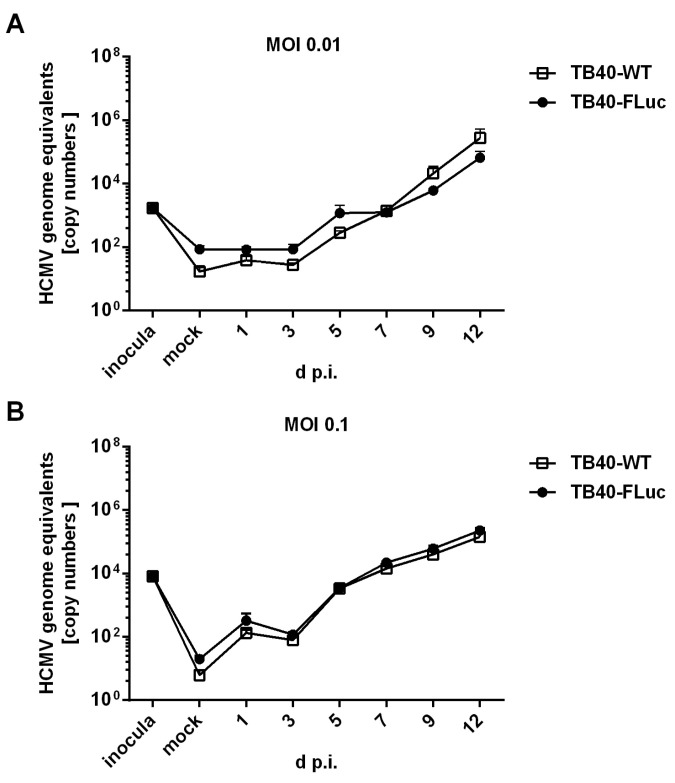
Comparison of viral replication kinetics between HCMV TB40-WT and TB40-FLuc. (**A**,**B**) HFFs were seeded in 12-well plates and infected with HCMV TB40-WT or TB40-FLuc at an MOI of 0.01 or 0.1. Viral supernatants were harvested on the indicated d p.i. The copy numbers of the HCMV genome equivalents were measured by HCMV-specific qPCR, and uninfected cells (mock) served as a negative control. The qPCR measurements were performed in sextuplicate and mean values ± SD are plotted.

In addition, viral replication kinetics were compared between HCMV TB40-WT and TB40-FLuc by performing HCMV-specific qPCR using media supernatant samples taken from infected HFFs in a temporal sequence of 1–12 d p.i. (Figure 6). At both MOIs of 0.01 (panel A) and 0.1 (panel B), the two viruses showed very similar courses of infection, with a pronounced increase in the copy numbers of viral genome equivalents between 3 and 12 d p.i. (Figure 6A,B, right half of the replication curves). No substantial differences were observed between HCMV TB40-FLuc and TB40-WT in this analysis, thus underlining the reliability of the FLuc reporter system.

### 3.5. Assessment of Antiviral Drug Activity in Two HCMV Reporter Systems (TB40-FLuc and AD169-GFP)

**Figure 7:**
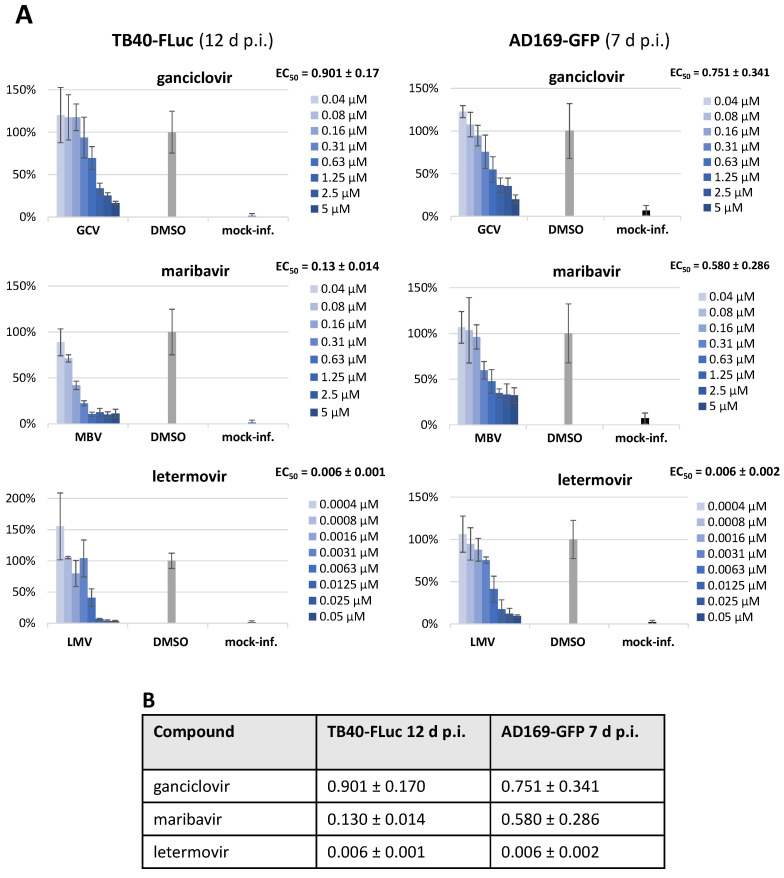
Antiviral effect of reference drugs GCV, MBV, and LMV for HCMV TB40-FLuc and AD169-GFP. HFFs were infected with either TB40-FLuc or AD169-GFP and treated with HCMV reference compounds in the stated concentrations. Antiviral activity was measured via quantitation of the fluorescence or luminescence signal at 7 d p.i. (AD169-GFP) or 12 d p.i. (TB40-FLuc), respectively (mean values of quadruplicates ± SD). (**A**) Primary data panels demonstrating antiviral drug activity; (**B**) summarized depiction of mean EC_50_ values ± SD (µM).

As a particularly relevant aspect of this infection reporter system, we performed an analysis of antiviral activity using a panel of anti-HCMV reference drugs. In all cases, the assessment, as carried out in a 96-well format using HFFs, was done in parallel with HCMV TB40-FLuc (Figure 7A, left) and AD169-GFP (Figure 7A, right). All reference compounds showed a clear concentration-dependent antiviral effect against both reporter viruses, with half-maximal effective concentrations (EC_50_) in the submicromolar (ganciclovir (GCV), maribavir (MBV)) or even nanomolar range (letermovir (LMV)). Notably, the EC_50_ values were very similar or did not show any indication of reporter-dependent deviation (Figure 7B). Thus, the HCMV TB40-FLuc reporter system proved to be very reliable and sensitive for the purpose of the fine quantitation of antiviral drug activities.

**Figure 8:**
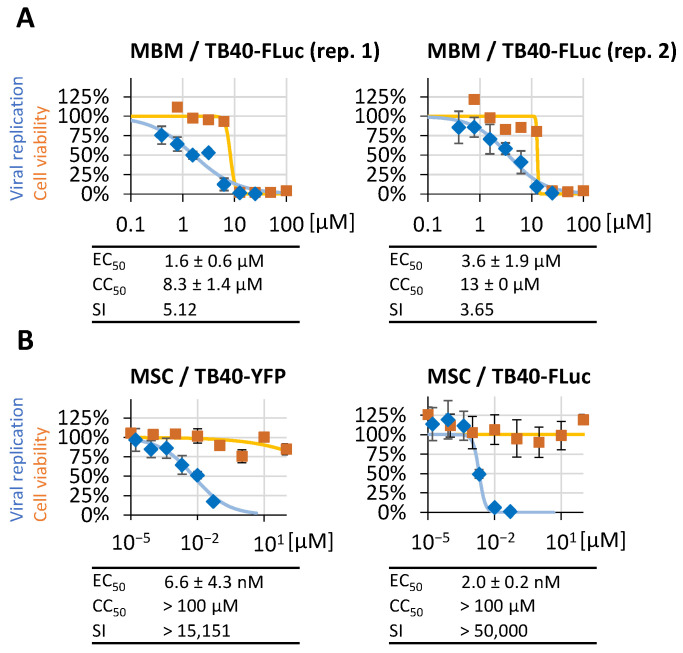
Assessment of antiviral activity of the autofluorescent inhibitor merbromin (MBM) and the host-directed antiviral MSC2530818 (MSC). HFFs were infected with TB40-FLuc or TB40-YFP, followed by MBM (**A**) or MSC (**B**) treatment, respectively, in serial dilutions as indicated. At 12 d p.i. (TB40-FLuc) or 7 d p.i. (TB40-YFP), luminescence- or fluorescence-dependent antiviral activity measurements were performed using the respective reporter signal (mean values of quadruplicates ± SD). (**A**) Two experimental replicates are shown for merbromin (panel A; rep. 1, left; rep. 2, right), demonstrating the appropriate reproducibility of the assay. (**B**) A comparison of TB40-FLuc with TB40-YFP is shown for MSC (panel B, right and left, respectively), demonstrating the consistency of the two assays.

In this context, the measurement of the anti-HCMV activity of autofluorescent compounds has posed a specific challenge in our previous studies. In particular, the use of HCMV reporter viruses expressing GFP, RFP, or similar fluorescence-based proteins was significantly restricted, as demonstrated for the autofluorescent inhibitor of viral nuclear egress activity, merbromin (MBM; [36,37]). To this end, the application of a non-fluorescent luciferase reporter offered additional opportunities, which were addressed by the HCMV FLuc system (Figure 8A). In this approach, the MBM treatment of HCMV-infected HFFs was evaluated by the TB40-FLuc measurements, thus confirming the previously reported antiviral potency of the drug in the low micromolar range, with EC_50_ values of 1.6 ± 0.6 µM and 3.6 ± 1.9 µM in two biological replicates of this experiment (Figure 8A, left and right panels, respectively). This antiviral activity was devoid of cytotoxicity, with clearly distinguishable CC_50_ values, as indicated by the SI values of 5.12 and 3.65, respectively. As we have analyzed a selection of direct-acting antivirals in Figure 7 and Figure 8A, we additionally intended to demonstrate the reliable assessment of host-directed antivirals with the use of the TB40-FLuc system. To this end, a developmental drug, MSC2530818, was analyzed for anti-HCMV activity by comparing the two reporter systems TB40-YFP (Figure 8B, left panel) and TB40-FLuc (right panel). The drug MSC targets the virus-supportive cyclin dependent kinase 8 (CDK8), and the pronounced anti-HCMV potential of CDK inhibitors, including the CDK8 inhibitor SEL120, has been described in our reports before [38]. Here, the novel drug candidate MSC comprises a very stringent anti-HCMV activity even at nanomolar concentrations, i.e., 6.6 ± 4.3 nM in the TB40-YFP system and 2.0 ± 0.2 nM in the TB40-FLuc system, respectively, also comprising very high in vitro SI values (as further characterized for MSC and other CDK8 inhibitors in detail elsewhere; Obergfäll, Wild, et al., 2024, Pharmaceutics, in preparation). These findings confirm that the TB40-FLuc reporter system can provide a very valuable input for antiviral drug research.

### 3.6. Determination of Reporter Sensitivity Comparing Different HCMV Infection Systems

**Figure 9:**
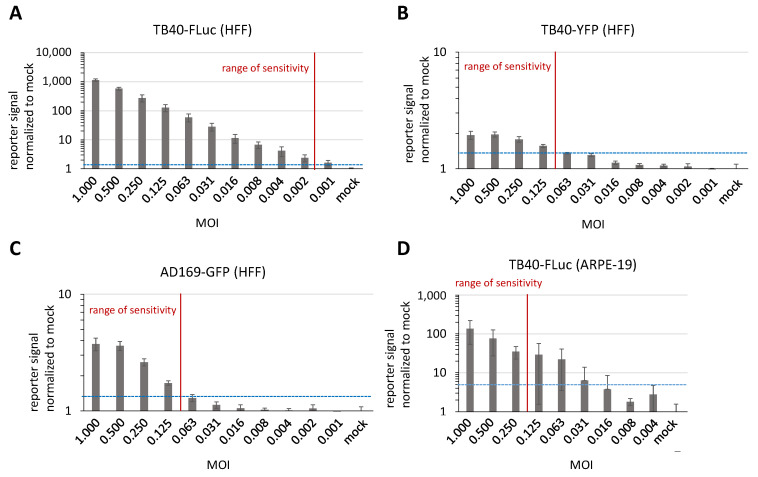
Increased reporter sensitivity of TB40-FLuc in comparison to other recombinant HCMVs. HFFs (**A**–**C**) or ARPE-19 cells (**D**) were infected with TB40-FLuc (**A**,**D**), TB40-YFP (**B**), or AD169-GFP (**C**) at a serial decrease of MOIs. For comparison of single-round infections, reporter signals of TB40 were analyzed at 4 d p.i. and GFP-fluorescence of AD169 at 3 d p.i. Each reporter signal was normalized to its background signal intensity (mock-infected). Mean values of determinations in quadruplicate ± SD are given. The mock sample value (plus 4-fold SD) was defined as the minimum of signal intensity to distinguish from the background (blue dashed line). The range of distinguishable signal intensities compared to the mock is marked as a vertical red line (range of sensitivity).

Finally, we addressed the question whether the HCMV TB40-FLuc system may have similar levels or even advantages compared to our previously established systems with TB40-YFP and AD169-GFP (Figure 9). For this purpose, HFFs were cultivated in 96-well formats and used for infection with one of the three reporter viruses. At the appropriate time points indicated, which were chosen as the typical duration of a single-round replication for these HCMVs, infected cells were harvested and subjected to measurements of the respective reporter signals. Importantly, the HCMV TB40-FLuc system proved to possess a substantially higher sensitivity in signal detection compared to the TB40-YFP and AD169-GFP systems (Figure 9, panels A, B, and C, respectively). The range of sensitivity was indicated by the lowest possible viral multiplicity of infection (MOI) that still produced a detectable viral reporter signal (marked by red vertical lines, in relation to the blue dotted horizontal lines giving the cut-off signal over the background). In order to address the question of whether the reporter virus may be adaptable to cell types, we then used HCMV TB40-FLuc for the infection of human retinal epithelial APRE-19 cells (Figure 9D). In this regard, we had to expect that the current stock virus of TB40-FLuc, which had been propagated on HFFs, only comprised a limited extent of the virion glycoprotein pentamer complex, i.e., that is normally responsible for conferring broad cell tropism. Nevertheless, this assay was also successful in showing serial gradation in reporter signals. Thus, the experiment demonstrated the principal capability of TB40-FLuc to infect epithelial cells, although the range of sensitivity was lower compared to that in HFFs (Figure 9A).

**Figure 10:**
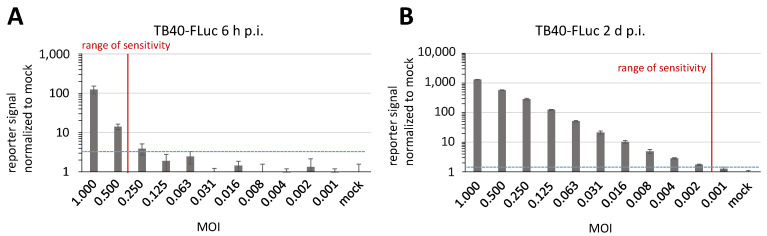
Detection of highly sensitive FLuc reporter activity at early time points during viral replication. HFFs were infected with TB40-FLuc at a serial decrease of MOIs. For comparison of different time points within the first replication cycle, the reporter signal was monitored at 6 h p.i. (**A**) and 2 d p.i. (**B**), and was also compared to 4 d p.i. (see Figure 9A). Each reporter signal was normalized to its background signal intensity (mock-infected). Mean values of determinations in quadruplicate ± SD are given. The mock sample value (plus 4-fold SD) was defined as the minimum of signal intensity necessary to distinguish from the background (blue dashed line). The range of distinguishable signal intensities compared to the mock is marked as a vertical red line (range of sensitivity).

As another parameter of sensitivity, the duration of infection with HCMV TB40-FLuc was varied from 4 d (Figure 9) to 2 d or 6 h p.i. (Figure 10A,B). Although in the example of measurement at 6 h p.i., the range of sensitivity still remained relatively low, the assay nevertheless already produced detectable values (as indicated by the rate-limiting MOI at a virus dilution factor between 0.500 and 0.250; Figure 10A). The sensitivity at 2 d p.i. was already very high (between 0.002 and 0.001; Figure 10B), and thus was basically indistinguishable from 4 d p.i., as shown in Figure 9A. Combined, these results emphasize the specific value and range of applicability of the HCMV TB40-FLuc reporter system.

### 3.7. Further Options for the Application of HCMV TB40-FLuc

In addition to the above-mentioned approaches, this study points to even more options for the application of HCMV TB40-FLuc that may have further value. One obvious advantage would be the comparison of different host cell types that may possess individual levels of susceptibility to HCMV infection and permissiveness for productive virus multiplication and release. Such experimental settings may allow for a characterization of this strain of HCMV in terms of cell type-specific tropism and virulence, and may even include so far poorly investigated minor target cell types such as those of the human placenta. Another topic of interest may address the use of recombinantly selected cell populations that show a knock-down or, alternatively, overexpression of virus-supportive host factors. Such aspects of virus–host interaction may be more easily studied by including specific reporter HCMVs with the infection such as the high-sensitivity reporter TB40-FLuc. Finally, the use of luciferase reporter activity in approaches to virus detection in situ or in vivo may open up further applications like HCMV organoid models, ex vivo tissue infection, and animal investigations. To this end, the established protocols for luciferase-based in vivo imaging [39,40,41] may provide a solid basis for applied procedures for pursuing HCMV analyses.

## 4. Conclusions

The present study demonstrates the generation and use of a novel reporter system based on the recombinant HCMV TB40-FLuc. The data illustrate the pronounced reliability and broad options for applicability offered by this HCMV recombinant. This was particularly emphasized by a number of viral characteristics that were found to be almost WT-like (e.g., titers of the produced stock viruses) or only deviating to a minor extent from comparable HCMVs (e.g., slightly delayed replication kinetics). Thus, these findings demonstrate that HCMV TB40-FLuc is employable in multiple settings, including primary human cells and other permissive cell types. Moreover, the reporter signal is easily quantifiable, while also showing an obvious advantage with its high reporter sensitivity. As an obvious limitation of the system, the necessity to prepare cell lysates for the measurement of luciferase activity should be taken into account. This step is not required when measuring an intracellular reporter signal, like GFP, but due to the fact that various HCMV reporters are available, as described here and elsewhere [42,43], a comparative mode of analysis appears to be optimal to achieve complementary results. Also in this study, a comparison was performed between HCMV TB40-FLuc and classical [16] or recently established reporter cytomegaloviruses [35,44]. A selection of five reporters, spanning the subfamilies of α-, β-, and γ-herpesviruses, demonstrated the broad usefulness of autofluorescence- and luciferase-based readout systems (Figure S1). In this quantitative assessment of approved reference drugs and developmental antiviral compounds, HCMV TB40-FLuc indicated its high reliability, thus representing a helpful option for confirmative and comparative analyses. In regard to cell-resident reporters [42,43], it should be emphasized that these can be highly valuable, and applicable to assay standardization, when used in the form of stably transfected, selected reporter cell lines. However, this point represents a general limitation when using primary cultures of non-immortalized cells, such as HFFs, which are restricted to a given range of low passages. In these cases, virus-encoded reporters may have specific advantages (Figure S1). Of note, the present study demonstrated the applicability of the reporter system in antiviral drug assessment, also including autofluorescent compounds, which may confer a specifically relevant benefit of the system. It should be mentioned, however, that this advantage only holds true as long as the analyzed compounds do not interfere with the luciferase activity per se, i.e., through artifactual FLuc assay interference. This possibility can be easily ruled out by performing enzymatic in vitro settings as a parallel control. Combined, we like to emphasize that even beyond the data presented in this study, a number of additional options for application appear directly open for establishing individual protocols. In essence, HCMV TB40-FLuc combines several molecular properties that can be utilized for the characterization of virus–host interaction, viral replication efficiency, HCMV-specific factors determining pathogenesis, antiviral drug assessment, and possibly even more so far unaddressed possibilities for HCMV research.

## Data Availability

The original contributions presented in the study are included in the article and Supplementary Materials, further inquiries can be directed to the corresponding author.

## Supplementary Materials

The following supporting information can be downloaded at: https://www.mdpi.com/article/10.3390/pathogens13080645/s1, Figure S1: Establishment of new antiviral reporter assays for α-, β, and γ-herpesviruses including their calibration with reference drugs.
