# Supplementary material for: Establishment of a Luciferase-Based Reporter System to Study Aspects of Human Cytomegalovirus Infection, Replication Characteristics, and Antiviral Drug Efficacy
Source: Pathogens. 2024 Jul 31;13(8):645. doi: 10.3390/pathogens13080645 (PMC11356942; doi:10.3390/pathogens13080645)
Supplement: Supplementary file 1 [file pathogens-13-00645-s001.zip › pathogens-3055532-supplementary.pdf]

pathogens

Supplementary Materials

Establishment of a Luciferase-Based Reporter System to Study Aspects of Human Cytomegalovirus Infection, Replication Characteristics, and Antiviral Drug Efficacy

Julia Tillmanns [1,‡], Jintawee Kicuntod [1,‡], Antonia Ehring [1,‡], Endrit Elbasani [2,§], Eva Maria Borst [2], Debora Obergfäll [1], Regina Müller [1] and Friedrich Hahn [1] and Manfred Marschall [1,*]

1 Institute for Clinical and Molecular Virology, Friedrich-Alexander University of Erlangen-Nürnberg (FAU), 91054 Erlangen, Germany; jul.tillmanns@fau.de (J.T.); jintawee.kicuntod@extern.uk-erlangen.de (J.K.); antonia.ehring@fau.de (A.E.); debora.obergfaell@fau.de (D.O.); mueller.regina@uk-erlangen.de (R.M.); friedrich.hahn@uk-erlangen.de (F.H.)
2 Institute of Virology, Hannover Medical School (MHH), 30625 Hannover, Germany; elbasani.endrit@mh-hannover.de (E.E.); borst.eva@mh-hannover.de (E.M.B.)
* Correspondence: manfred.marschall@fau.de; Tel.: +49-9131-85-36096
§ Current address: Immuno-Oncology, Oncology Research, Orion Corporation, 20360 Turku, Finland.
‡ These authors contributed equally to this work.

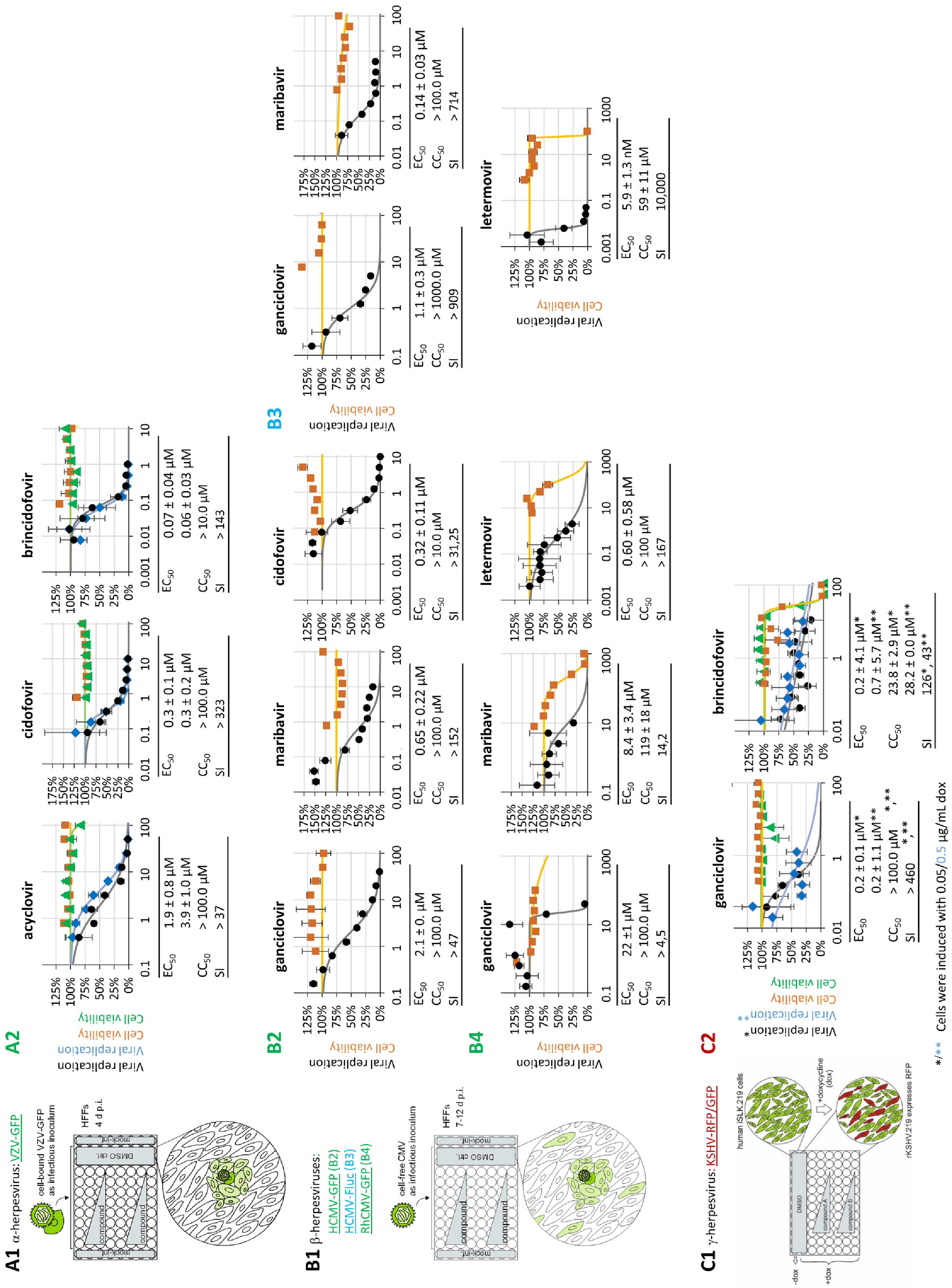
A1 α-herpesvirus: VZV-GFP
cell-bound VZV-GFP as infectious inoculum
HFFs 4 d p.i.
mock-inf.
DMSO ctrl
compound
A2
Viral replication
Viral replication
Cell viability
Cell viability
acyclovir
EC50 1.9 ± 0.8 µM
3.9 ± 1.0 µM
CC50 > 100.0 µM
SI > 37
cidofovir
EC50 0.3 ± 0.1 µM
0.3 ± 0.2 µM
CC50 > 100.0 µM
SI > 323
brincidofovir
EC50 0.07 ± 0.04 µM
0.06 ± 0.03 µM
CC50 > 10.0 µM
SI > 143
B1 β-herpesviruses:
HCMV-GFP (B2)
HCMV-Fluc (B3)
RhCMV-GFP (B4)
cell-free CMV as infectious inoculum
HFFs 7-12 d p.i.
B2
ganciclovir
EC50 2.1 ± 0. µM
CC50 > 100.0 µM
SI > 47
maribavir
EC50 0.65 ± 0.22 µM
CC50 > 100.0 µM
SI > 152
cidofovir
EC50 0.32 ± 0.11 µM
CC50 > 10.0 µM
SI > 31,25
B3
ganciclovir
EC50 1.1 ± 0.3 µM
CC50 > 1000.0 µM
SI > 909
maribavir
EC50 0.14 ± 0.03 µM
CC50 > 100.0 µM
SI > 714
letermovir
EC50 5.9 ± 1.3 nM
CC50 59 ± 11 µM
SI 10,000
B4
ganciclovir
EC50 22 ± 1 µM
CC50 > 100.0 µM
SI > 4,5
maribavir
EC50 8.4 ± 3.4 µM
CC50 119 ± 18 µM
SI 14,2
letermovir
EC50 0.60 ± 0.58 µM
CC50 > 100 µM
SI > 167
C1 γ-herpesvirus: KSHV-RFP/GFP
human iSLK.219 cells
+doxycycline (dox)
rKSHV.219 expresses RFP
-dox
+dox
DMSO
compound A
compound B
C2
Viral replication*
Viral replication**
Cell viability
Cell viability
ganciclovir
EC50 0.2 ± 0.1 µM*
0.2 ± 1.1 µM**
CC50 > 100.0 µM *,**
SI > 460 *,**
brincidofovir
EC50 0.2 ± 4.1 µM*
0.7 ± 5.7 µM**
CC50 23.8 ± 2.9 µM*
28.2 ± 0.0 µM**
SI 126*, 43**
*/** Cells were induced with 0.05/0.5 µg/mL dox

Figure S1. Establishment of new antiviral reporter assays for α-, β, and γ-herpesviruses including their calibration with reference drugs. The virus recombinants **(A)** VZV Oka-GFP (α-herpesvirus), **(B)** HCMV AD169-GFP, HCMV TB40-FLuc, rhesus monkey cytomegalovirus RhCMV-GFP (β-herpesviruses), and **(C)** rKSHV.219-GFP/RFP (γ-herpesvirus) were used for the infection of HFFs (VZV, HCMV, RhCMV) or were continuously passaged in iSLK.219 carrier cells (KSHV), respectively. **(A1, B1, C1)** Schematic depiction of the assay systems, performed in a 96-well plate format. Notably, all these assays can be performed in the 96-well format, which allows to work with very small quantities of material, or alternatively in larger well formats, such as the 12-well format, which may increase signal intensities. For the VZV-GFP system, virus stock VZV Oka-GFP was applied as a cell-bound infectious inoculum. For the HCMV and RhCMV systems, virus stock was applied as a cell-free infectious inoculum (i.e. either in the form of media supernatant samples directly harvested from CMV-infected HFF producer cultures, or alternatively ultracentifugation-purified virus concentrates). After a 90 min period of inoculum adsorption to the cell surfaces, antiviral compounds were added to the culture media at serial dilutions (DMSO served as an infection-solvent control; mock-infected cells served as a negative control). For the KSHV-GFP/RFP system, in iSLK.219 cells lytic replication of KSHV (RFP reporter signal) was induced by the addition of 0.05 or 0.5 μg/ml doxycycline (dox) to the culture media. Cultivated dox-induced iSLK.219 cells were treated with antiviral compounds added at serial dilutions (DMSO served as a positive-infection solvent control; dox-untreated mock cells served as a negative control). HFFs or iSLK.219 cells were harvested at 4 d p.i. (VZV), 7-12 d p.i. (CMVs), or 1 d after treatment (KSHV), respectively. Samples were fixed by a 10 min incubation with 10% formalin, washed with PBS, and used for quantitation of the GFP or RFP reporter signal by either automated fluorometry (Victor X4 Multimode Plate Reader, Perkin Elmer, Waltham, MA, USA), or PicoMD counting (ImageXpress® Pico device, Molecular Devices LLC, San Jose, CA, USA), and data evaluation using CellReporterXpress® software (version 2.9.3.1183, Molecular Devices LLC) as described earlier [29]. **(A2, B2, B3, B4, C2)** The antiviral activity of reference drugs against the panel of viruses was determined as indicated. EC_{50} values refer to measurements in quadruplicate or triplicate. Mean values ± SD of either two parallel experimental replicates, or one representative replicate taken out of multiple determinations, are given.
